# The effects of kettlebell training versus resistance training using the own body mass on physical fitness and physiological adaptations in obese adults: a randomized controlled trial

**DOI:** 10.1186/s13102-024-00894-6

**Published:** 2024-05-07

**Authors:** Karuppasamy Govindasamy, Hemantajit Gogoi, Nidhal Jebabli, Sultan Mansour Bediri, Mohammed Aljahni, Koulla Parpa, Cain C. T. Clark, Urs Granacher, Hassane Zouhal

**Affiliations:** 1 Department of Physical Education and Sports Sciences, Faculty of Science and Humanities, SRM Institute of Science and Technology, Kattankulathur, Chennai, Tamilnadu India; 2 Department of Physical Education, Rajiv Gandhi University, Itanagar, Arunachal Pradesh 791112 India; 3 Research Unit: Sport Sciences, Health and Movement, Higher Institute of Sport and Physical Education of Kef, UR22JS01, University of Jendouba, Kef, 7100 Tunisia; 4 Department of Physical Education, Al-Bahah University, Al Bahah, Saudi Arabia; 5 College of Arts and Humanities, Department of Educational Sciences, Jazan University, Jazan, 45142 Saudi Arabia; 6 Department of Sport and Exercise Science, UCLan University of Cyprus, Pyla, Cyprus; 7 Institute for Health and Wellbeing, Coventry University, Coventry, CV1 5FB UK; 8 College of Life Sciences, Birmingham City University, Birmingham, B15 3TN UK; 9 Department of Sport and Sport Science, Exercise and Human Movement Science, University of Freiburg Sandfangweg 4, 79102 Freiburg, Germany; 10 Univ Rennes, M2S (Laboratoire Mouvement, Sport, Santé), EA 1274, Rennes, F-35000 France; 11 Institut International des Sciences du Sport (2IS), Irodouer, 35850 France

**Keywords:** Strength training, Exercise training, Obesity, Overweight, Endurance

## Abstract

**Aim of study:**

This study aimed to explore the effects of different types of resistance training using kettlebells versus the own body mass, in comparison to a passive control, on key physical fitness and physiological parameters in young, obese adults.

**Methods:**

Data from 60 sedentary, obese male college students, aged 17–26, were used for final analyses. Participants were randomly assigned to one of three groups: a control group (CG, *n* = 20, no training), a kettlebell resistance training group (KRTG, *n* = 20), or a bodyweight resistance training group (BWRTG, *n* = 20). Selected measures of physical fitness were tested using the 12-minutes run test, the push-up test, the sit-up test, and the sit-and-reach test. Physiological measures included vital capacity, resting and maximum heart rate (HRmax), mean arterial blood pressure, breath holding time, and respiratory rate. Biochemical variables were measured in the morning, in a fasted state, and comprised high and low density lipoprotein, total cholesterol, and triglycerides. The 12-weeks progressive KRTG and BWRTG were specifically tailored using sets, repetitions, and intensity levels.

**Results:**

Notable findings include significant body fat reductions in BWRTG (*p* < 0.001; d = 1.53) and KRTG (*p* < 0.001; d = 1.43), and a substantial increase in VO2max for BWRTG (*p* < 0.001; d = 1.32) and KRTG (*p* < 0.001; d = 1.34) compared to CG. KRTG also showed significant improvements in vital capacity (*p* < 0.001; d = 1.61) and reductions in resting heart rate (*p* = 0.024, d = 1.05) and respiratory rate (*p* = 0.001, d = 1.55), with BWRTG showing similar trends (resting heart rate: *p* = 0.041, d = 1.35; respiratory rate: *p* = 0.001, d = 1.98). Both intervention groups significantly improved breath holding time (KRTG: *p* = 0.001, d = 1.58; BWRTG: *p* < 0.001, d = 1.98) and reduced total cholesterol and low-density lipoprotein levels compared to CG.

**Conclusions:**

This study demonstrates that both KRTG and BWRTG are effective in improving body composition and selected fitness and physiological measures. Thus, resistance training using kettlebells or bodyweight training are recommended if the goal is to improve body composition and fitness in obese male adults.

**Trial Registration:**

OSF, September, 28th 2023. 10.17605/OSF.IO/Z6Y9Gosf.io/2mb98

**Supplementary Information:**

The online version contains supplementary material available at 10.1186/s13102-024-00894-6.

## Introduction

Obesity rates have risen progressively over the last few decades and are currently at record-high levels, irrespective of age, sex, race, and smoking status [1]. The obesity pandemic is particularly severe in developing nations [1, 2], and is considered a disease consisting of multiple aetiologies (i.e., genetics and psychosocial factors) [3].

Cross-sectional and longitudinal studies have shown increasing trends towards physical inactivity and a sedentary lifestyle over the past few decades [4]. An increase in sedentary time can result in increased body fat (BF), which is a significant public health concern due to it’s irrefutable association with several non-communicable disease, including diabetes, cardiovascular disease, and multiple cancers [5]. Interestingly, it has been demonstrated that body-mass-index (BMI) increases are strongly and independently related to impaired cardiorespiratory function and physical fitness [6].

Evidence from recent systematic reviews and meta-analyses have emphasized the importance of resistance training for health-related outcomes through increased muscle mass and strength including a reduction of several risk factors of cardiovascular disease, type 2 diabetes, obesity, cancer and mortality [7, 8]. Regarding obesity, Miller et al. [9] specified that only 12-weeks of body weight resistance training, including squats and push-ups, led to increased muscular strength, flexibility, and cardiovascular fitness and decreased body mass, fat percentage, and waist circumference in overweight and obese adults. There is evidence that resistance training promotes muscular hypertrophy through enhanced muscle protein synthesis [10]. This process involves the increase in muscle mass due to the stimulation of muscle protein synthesis, resulting in greater gains in muscle size [10]. Resistance training related increases in muscle mass improve basal metabolic rates and insulin sensitivity by optimizing glucose uptake in obese adults [10]. This is achieved through increased GLUT4 translocation, which enhances glucose uptake into muscle cells [10]. These improvements in insulin sensitivity and optimization in glucose uptake mitigate the risk of type 2 diabetes in obese individuals.

However, the question remains open as to the most effective type of resistance training to improve the above described adaptive processes. In fact, body mass resistance exercises can enhance muscular strength and cardiovascular fitness through the activation of multiple muscle groups, promoting muscle protein synthesis [11]. When performing body mass exercises, multiple muscle groups are engaged, leading to an increase in muscle protein synthesis and subsequent improvements in muscular strength and cardiovascular fitness [11]. Body mass exercises also improve functional capacity by stimulating neural adaptations and increasing motor unit recruitment [11]. Moreover, body mass exercises, when performed regularly, can increase basal metabolic rate and improve lipid metabolism, leading to better body mass management and metabolic health outcomes [11], ultimately improving overall physical fitness.

While body weight resistance training offers benefits like increased strength and flexibility, it has certain constraints. Indeed, progressive overload is limited by one’s own body mass, which can restrict muscle growth over time. Some muscles may be under-targeted, leading to imbalances, and significant muscle hypertrophy is more difficult to achieve compared to using machines or free weights. Over time, regular body mass exercises can result in plateaus in strength gains, and variation can be limited, potentially reducing motivation. Combining body mass routines with other types of weight-bearing exercises can help address these limitations.

Kettlebell training is a popular resistance training type that received a lot of attention over recent years [12]. Kettlebell training is suitable for the performance of ballistic full-body movements with the usage of a cannonball-shaped iron object. The character of the kettlebell exercises demands high and full body muscle activations in all planes with a large range of motion [13]. This dynamic and explosive exercise stimulus promotes physical fitness, including muscle strength and power, flexibility, and even cardiorespiratory fitness [13, 14]. Moreover, kettlebell training improved aerobic capacity due to a large cardiovascular demand in healthy individuals [15]. In addition, Otto et al. [16] indicated that kettlebell training significantly enhanced measures of muscular strength (e.g., back squat) and power (e.g., vertical jump height) in healthy males.

Kettlebell exercise can therefore elicit cardiovascular, neuromuscular, and metabolic responses to enhance measures of muscle strength and aerobic capacity [13–16].

Despite the well-documented benefits of different training modalities in various populations, the specific comparison of the effects of kettlebell training and body mass resistance training has not yet been sufficiently addressed for obese populations in the scientific literature. Therefore, this study aimed to fill this gap by providing empirical evidence on the effectiveness of these distinct resistance training modalities in inducing specific physical fitness and physiological adaptations in male obese adults. By doing so, this study sought to evaluate the effects of kettlebell resistance training (KRTG) versus resistance training using the own body mass (BWRTG) and passive control on selected measures of physical fitness, body composition, and physiological responses in obese male adults. Based on the relevant literature [15–17], we hypothesized that both exercise types would improve physical fitness and physiological adaptations compared to a control with larger effects following KRTG compared with BWRTG.

## Methods

### Participants

To determine the required sample size, an a priori power analysis was calculated using G*Power (version 3.1.9.2, University of Kiel, Kiel, Germany) with an assumed power of 0.90 an alpha level = 0.01 and an effect size measure Cohen’s f = 0.31 (i.e., VO2max) based on the outcome of a related study on the effects of high intensity intermittent functional training on VO2max in young obese adults [18]. The analysis revealed that a total sample size of *N* = 45 would be sufficient to achieve medium-sized group-by-time interactions. Moreover, to account for loss to attrition, we recruited additional participants considering the adoption of exercise programs in currently inactive individuals (*N* = 76).

In India, the consensus guidelines defined overweight as those with BMI between 23.0 and 24.9 kg/m^2^ and obesity as those having BMI ≥ 25.0 kg/m^2^ [19]. Accordingly, a total of 76 obese, sedentary male college students aged 17– 26 years volunteered to participate in this study. Inclusion criteria were a priori defined and comprised of BMI > 25 kg/m^2^, non-smoking, no cardiovascular diseases including diabetes, no known incidence of liver dysfunction, renal impairment, an endocrine disorder, or weight-loss pill consumption, and not undertaking systematic habitual physical training (> one session per week). Five participants were excluded from the study because they did not meet the inclusion criteria.

**Fig. 1:**
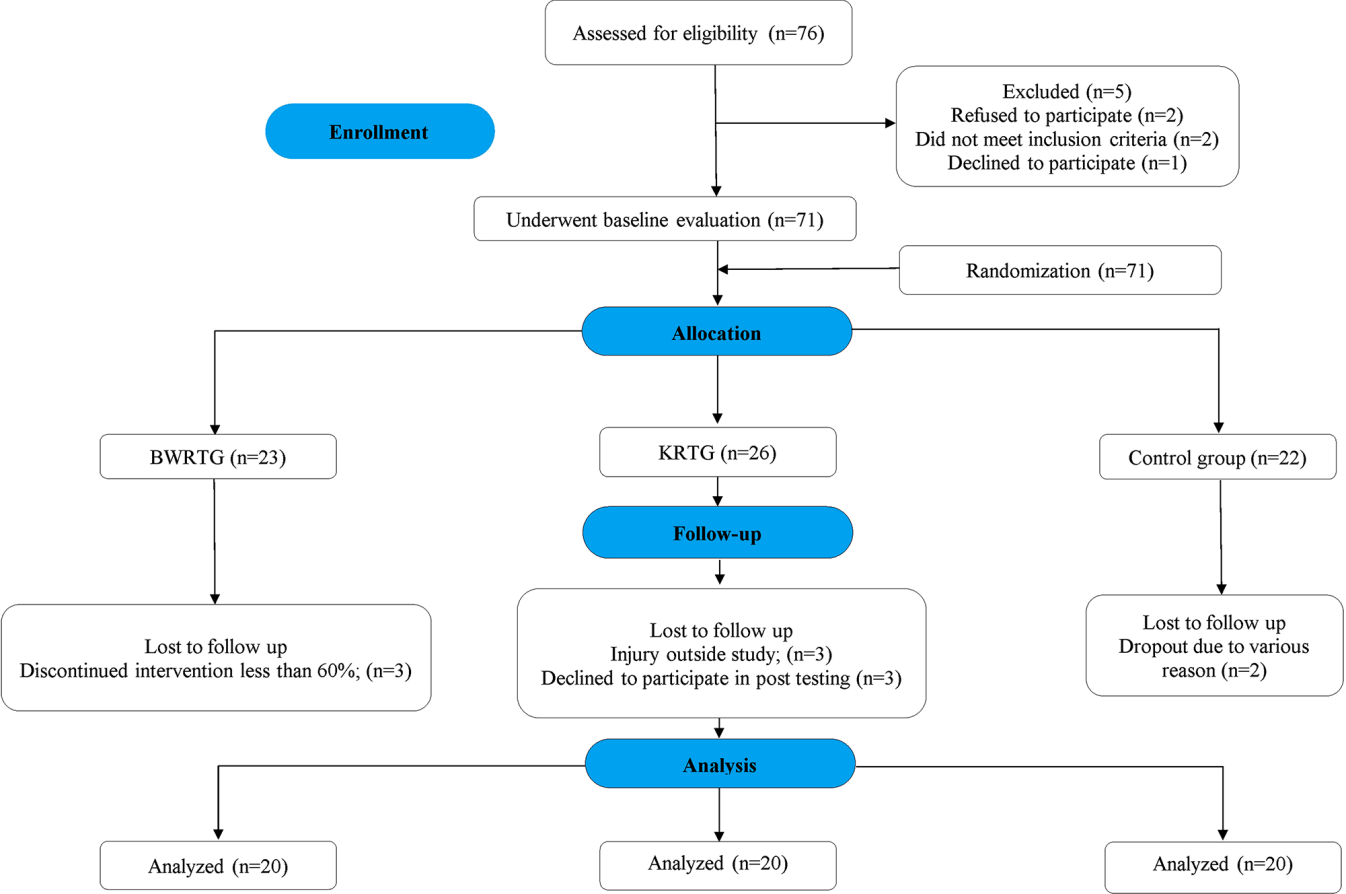
Study flow chart. Body weight resistance training training (BWRTG), kettlebell resistance training (KRTG), and the passive control group (CG)

In our study, participants underwent a detailed initial screening process, after which they were allocated to one of three groups: Body weight resistance training group (BWRTG, *n* = 23), kettlebell resistance training group (KRTG, *n* = 26), or a passive control group (CG, *n* = 22). The randomized allocation of the study participants to the experimental groups was achieved using a computer-generated random number sequence with MS Excel. By doing so, we aimed to maintain the integrity of the random assignment process. Finally, 60 participants completed the program and were included in the data analysis (KRTG: *n* = 20; BWRTG: *n* = 20; CG: *n* = 20). The exclusion of 16 participants was due to their inability to complete the training program for various personal reasons, including time constraints, logistical challenges in attending the training center, and loss of interest in the program. While we recognize that this deviates from the intention-to-treat analysis framework, we believe it was necessary to ensure the accuracy and reliability of our data. This exclusion has been carefully considered in the context of the study’s internal validity, and we have already taken measures to mitigate participant’s attrition from negatively impacting on the trial’s outcomes. The Fig. 1 further explains the flow of the study.

The experimental protocol was registered (July 28th, 2022) and approved by the institutional review board of SRM Medical College Hospital & Research Centre (8484/IEC/2022), and informed consent was obtained from each participant after risks and benefits were explained. The study was in accordance with the latest version of the Declaration of Helsinki and adheres to CONSORT guidelines.

### Procedures

Two familiarization sessions were scheduled one week before the baseline assessment to acquaint participants with the applied physical fitness tests.

Participants were also advised to maintain their normal nutritional routines throughout the testing period. Across the intervention period, three test sessions were scheduled with a rest period of at least 48 h between the sessions. The 12-Minute run test, the push-up, sit-up, and sit-and-reach tests were recorded on the first test day. Physiological measurements (e.g., VO2max, vital capacity, resting and maximal heart rate, mean arterial blood pressure, breath holding time, respiratory rate) were taken on the second day. Biochemical measurements (e.g., fasting glucose, total cholesterol, low-density lipoprotein cholesterol, high-density lipoprotein cholesterol, and triglycerides) were recorded on the third test day. The order of the tests was the same for each participant and test time point. Before each test session, participants performed a standardized 10-min warm-up, including low intensity running or cycling, dynamic stretching, and dynamic exercises.

### Exercise interventions

The intervention programs spanned 12 weeks and comprised three weekly sessions, each lasting 60 min. Exercise progression of each program was controlled through the involved strength and conditioning specialists. The passive control group did not engage in any prescribed exercise during the study. The training interventions were designed and administered by expert strength and conditioning coaches from the Fitness Center of the SRM Institute of Science and Technology. We maintained a participant-to-coach ratio of 5:1 to ensure personalized attention and effective training. Each training session was structured into three phases: a 10 min warm-up, the main exercise program lasting 50-min and including a cool-down period. The warm-up and cool-down phases comprised low-intensity running or cycling, and dynamic stretching.

### Body mass resistance training

For the BWRTG, the training program primarily utilized the participants’ own body mass, incorporating a variety of exercises such as marching on the spot, side leg raises, climbers, half jacks, jumping jacks, basic burpees, high knees, squats, squat jab, sprinter lunges, and push-ups with jab-cross. For each exercise, 3–5 sets with a duration per exercise of 20 s separated by 1 min of rest between sets, with at least a 5 min recovery between each exercise were scheduled. The progression of the exercises, over 12 weeks of training, was carried out gradually depending on the complexity of the tasks and their intensity. A detailed outline of a typical BWRTG training session can be found in Table 1. Previous research showed that resistance training using the own body mass was effective to enhance muscular strength and sport-specific performance [20].

**Table 1:** Exemplified exercise session for body weight resistance training (BWRTG) taken from week three of the exercise program

Sequence	Exercise	Sets	Repetitions/Duration	Rest between exercises
Warm-up	Gentle marching on the spot	1	2 min	-
	Arm circles	1	1 min	-
	Leg swings	1	2 min	-
	Dynamic chest stretches	1	1 min	-
	Gentle butt kicks	1	1 min	-
Main workout	Marching on the spot	3	20 s	15 s
	Side leg raises	3	20 s	15 s
	Climbers	3	20 s	15 s
	Half jacks	3	20 s	2 min
	Jumping jacks	3	20 s	15 s
	Basic burpees	3	20 s	15 s
	High knees	3	20 s	15 s
	Squats	3	20 s	15 s
	Squat jab	3	20 s	15 s
	Sprinter lunges	3	20 s	15 s
	Push-ups with jab cross	3	20 s	2 min (between sets)
Cool down	Hamstring stretch	1	30 s	-
	Quadriceps stretch	1	30 s	-
	Calf stretch	1	30 s	-
	Arm and shoulder stretch	1	30 s	-
	Spinal twist stretch	1	30 s	-
	Deep breathing and relaxation	1	2–3 min	-

Training intensity (60–80% of HRmax) was controlled using a heart rate monitor (Polar T31, Kempele, Finland) (Grace et al., [26])

**Table 2:** Exemplified exercise session for kettlebell resistance training (KRTG) taken from week three of the exercise program

Sequence	Exercise	Sets	Repetitions/Duration	Rest between exercises	Rest between sets
Warm-up	Dynamic stretching	1	5 min	-	-
	Kettlebell swings	1	1 min	-	-
Main workout	Side lunge	3	10 reps	15 s	1 min
	Curtsy squats	3	10 reps	15 s	1 min
	Straight arm sit ups	3	10 reps	15 s	1 min
	Single arm biceps curls	3	10 reps (each side)	15 s	1 min
	Triceps extensions	3	10 reps	15 s	1 min
	Turkish getups	3	5 reps (each side)	15 s	1 min
	Squat with single arm press	3	10 reps	15 s	1 min
	Overhead Squat	3	10 reps	15 s	1 min
	Overhead to knee tuck	3	10 reps	15 s	1 min
	Pushups on the kettlebell	3	10 reps	15 s	1 min
	Kettlebell windmills	3	10 reps (each side)	15 s	1 min
Cool down	Static Stretching	1	5 min	-	-
	Deep breathing	1	2–3 min	-	-

Training intensity (60–80% of HRmax) was controlled using a heart rate monitor (Polar T31, Kempele, Finland) (Grace et al., [26])

### Kettlebell training

The KRTG comprised various exercises such as side lunges, curtsy squats, straight arm sit-ups, single arm biceps curls, triceps extensions, Turkish getups, squats with single arm press, overhead squats, overhead to knee tucks, push-ups on the kettlebell, kettlebell windmills. KRTG was conducted at an intensity of 60–80% of the 1 repetition maximum (1-RM). The 1-RM was determined using the Kettelbal 1-RM calculator (https://kettlebellexercises.fitness/online-calculators/kettlebell-1rm-calculator/). A series of kettlebell weights (8, 12, 16, 20, 24 and 32 kg) were used. Each training session ranged from basic to advanced exercise levels.

For KRTG specifically, the progression pattern included a gradual increase in exercise complexity and intensity every four weeks. This was achieved by varying exercise types from basic to advanced, increasing resistance levels (*kettlebell* mass) and adjusting speed of execution.

In fact, eleven exercises were completed in each training session. Each exercise was performed in 3–5 sets of 5–10 repetitions, with one minute rest between sets with at least a 5 min recovery between each exercise. The resistance load changed according to each person’s individual physical abilities (%1-RM). In other words, the first four weeks focused on basic functional movements with a low resistance load (60% of 1-RM), the next four weeks introduced a moderate resistance load (70% of 1-RM) and the last four weeks focused on high resistance load exercises (80% of 1-RM).

The KRTG was planned, conducted, and supervised according to the recommendations of the American College of Sports Medicine [21].

### Testing

In our study, we employed a non-blinded assessment approach, where assessors were informed of the participants’ group allocations. This methodology was selected to align with the distinct nature of each intervention and to streamline the logistical aspects of the study. To enhance the reliability of our findings, we rigorously implemented standardized assessment protocols across all groups, ensuring consistency and objectivity in our evaluations. Furthermore, the primary outcomes of our study were based on objective measurements, which inherently minimize the risk of bias. The details of testing procedure are mentioned below Table 2.

### Anthropometry

On an electronic scale, participants’ body mass was assessed to the nearest 0.01 kg while wearing minimal clothes and unshod (Seca, Hamburg, Germany). Body height was measured to the nearest 0.1 cm with a stadiometer (Seca, Hamburg, Germany) according to standardized procedures described elsewhere [22–24]. The body mass index (BMI) was calculated as body mass (kg)/height^2^ (m).

### Cardiovascular fitness

Cardiovascular fitness was tested using the 12-Minute Cooper run test [25]. Cooper’s standardized equation was then used to convert the distance run to an estimate of VO2max (ml·kg^− 1^·min^− 1^) = (22.351 x distance covered in kilometers) − 11.288 [25]. The heart rate was measured using short-range radio telemetry (Polar T31, Kempele, Finland) [26].

### Muscular endurance

Participants performed the muscular endurance test in prone position on a mat with their hands placed under their shoulders, their fingers stretched out, their legs straight, parallel, and slightly apart, and their toes tucked under. The participants pushed up from the mat with their arms until the arms were straightened out, keeping their legs and back straight. The participants then lowered their bodies using their arms until their elbows were bent at a 90-degree angle and their upper arms were parallel to the floor. Participants were instructed to perform as many 90° push-ups as possible following a specified cadence signalled by “down” and “up.” The subjects were stopped when they made the second mistake. The following technical performance items were categorized as “mistakes”: knees touching the floor, upper or lower back swaying, failing to fully extend the arms, or bending to 90 degrees at the elbow. Their score was the number of 90° push-ups that were correctly performed [27].

A second test for the assessment of muscular endurance was the sit-up test that measures trunk muscular endurance (BS-SU, Inbody, Seoul, Republic of Korea) [28]. To help participants understand the procedure, two familiarization trials were allowed with the help of assistants who informed them of the number of repetitions during the measurement.

### Flexibility

The sit and reach test was performed in accordance with the test procedures as described by Gea-García et al. [29]. Lower body and trunk flexibility was assessed in a sitting position with the legs straight while attempting to reach forward as far as possible. A sit-and-reach box was used to record the distance reached by the hand in centimeters [29].

### Body composition

Body height (cm), mass (kg), BMI, and body fat percentage were measured using a standing stadiometer and a bioimpedance system (BSM 330, Inbody, Seoul, Republic of Korea) [30]. Participants were classified as average with a BMI < 23, overweight with a BMI ≥ 23, and obese with a BMI ≥ 25 [30]. Obesity was defined as BMI ≥ 25 kg/m^2^, according to the Asia Pacific standards of the WHO guidelines [30].

### Vital capacity

The respiratory parameters were measured using the CSMI Spirometrics instrument (Scott Medical, Antrim, Northern Ireland). This test sought to capture the maximum air volume that is expired after a forceful, rapid, and deep expiration, the forced vital capacity (FVC), and was recorded in liters [3].

### Resting and maximal heart rate

The measurement of resting heart rate or pulse rate (the number of heartbeats per minute) was taken after a few minutes upon waking whilst still lying in bed. Resting heart rate was obtained following 10 min of supine rest, and the maximal heart rate was measured during the Cooper’s 12-Minute Run using a heart rate monitor (Polar T31, Kempele, Finland) [26].

### Mean arterial blood pressure

Before undertaking a manual or automated MABP measurement, participants had to be lying in a supine position on a bed with legs uncrossed. Prior to testing, participants were kindly asked to relax for at least five minutes [31].

### Breath holding time

Participants were asked to make a full exhalation followed by a deep inhalation and then hold their breath for as long as possible.

### Respiratory rate

Manual counts were used as respiratory reference rates. To ensure consistency and eliminate variations, a single dedicated and trained medical staff member was deployed to observe and manually count respiratory rates. Electronic and manual recordings were started concurrently for every participant. From the electronic recordings, respiratory rates were calculated at the exact same 60 s as when manual recordings were recorded. They were then benchmarked for comparative analysis. Hospital medical staff reported all manual counts and diagnoses on case report forms.

### Blood sample analysis

Following a 12-hour overnight fasting period, venous blood samples (15 mL) were obtained from an antecubital vein with participants in a sitting position, after a 20-minute rest, between 07:00 a.m. and 09:00 a.m. at baseline and week 12. The blood was immediately transferred into vacutainer tubes (Becton Dickinson, Rutherford, NJ, USA) containing 0.1% EDTA as the anticoagulant for estimating the hematological status. Serum or plasma was separated through centrifugation at 2500 rpm for 15 min at 4 °C and then stored at − 80 °C until analysis. Hematological entities, including fasting glucose, total cholesterol (TC), low-density lipoprotein cholesterol (LDL-C), high-density lipoprotein cholesterol (HDL-C), and triglycerides (TG) were measured using an automated biochemical analyzer [32].

### Statistical analyses

Data were reported as means and standard deviations (± SD). The normality of all variables was tested and confirmed using the Kolmogorov-Smirnov procedure, whilst the Levene’s test was used to determine the homogeneity of variance. Test-retest reliability was assessed for all tests using intra-class correlation coefficients (ICCs) and coefficients of variation (CV) [33] only for control group. Training-related effects were evaluated using a linear mixed effects model which was operationalized in the form of an analysis of variance (ANOVA) with repeated measures (3 groups x 2 times). In case of significant group-by-time interactions, a Bonferroni adjusted post hoc test was performed. Greenhouse-Geisser corrections were used when the assumption of sphericity (Mauchly’s test) was violated. Partial eta squared (ηp^2^) were taken from ANOVA output, and Cohen’s d effect sizes (d) were calculated to quantify meaningful differences in the data with demarcations of trivial (< 0.2), small (0.2–0.59), medium (0.60–1.19), large (1.2–1.99), and very large (≥ 2.0) [34]. We also conducted a predefined contrasts analysis (Abdi & Williams, n.d.) to test the following hypothesis, H1, specifically any training condition (e.g., BWRTG, KRTG) would yield greater improvements in the outcome measures than the control group. Accordingly, we compared the control condition vs. BWRTG and KRTG (coded as CG, BWRTG, and KRTG, respectively). This approach compared one (or more) condition(s) vs. the grand mean of the specified contrasts. Indeed, post-hoc analysis, while useful, does not yield sufficient insight into multiple levels or detailing patterns in response; contrast analyses allow researchers to test theory-driven expectations directly against empirically derived group or cells means [35, 36]. Statistical significance was set *a priori¸* at *p* < 0.05. The statistical analysis was carried out using Statistica Version 13.2 software (StatSoft, France) and R (R Core Team (2018). R: A Language and environment for statistical computing. [Computer software]. Retrieved from https://cran.r-project.org/) using the *Car: Anova* package [37]; car: Companion to Applied Regression. [R package]. Retrieved from https://cran.r-project.org/package=car).

## Results

All participating individuals received treatment as allocated. No training or test-related injuries occurred. Absolute and relative test-retest reliability measures (intra-class correlation coefficients [ICC]) for the assessed tests ranged from 0.54 to 0.99, while coefficients of variation (CV) ranged from 1.6 to 12.2% (Table 3).

**Table 3:** Intraclass correlation coefficients (ICCs) for relative reliability and coefficients of variation for absolute reliability of the applied physical fitness, physiological and biochemical tests

Variables	ICC	95%CI	CV (%)
Fitness variables			
VO2max (ml/kg/min)	0.55	0.527–0.604	12.2
Push-Ups (numbers)	0.54	0.170–0.817	10.1
Sit-Ups (numbers)	0.85	0.617–0.940	5.4
Sit and Reach (centimeters)	0.79	0.715–0.821	10.7
**Physiological variables**			
Vital Capacity (mL)	0.90	0.734–0.958	9.1
Resting Heart Rate (bpm)	0.93	0.821–0.972	2.3
Mean Arterial Blood Pressure (mmHg)	0.7	0.412-0812	6.8
Breath Holding Time (s)	0.55	0.488–0.883	13
Respiratory Rate (numbers)	0.93	0.822–0.972	6.6
**Biochemical variables**			
High Density Lipoprotein (HDL) (mg/dl)	0.67	0.567–0.869	5
Low Density Lipoprotein (LDL) (mg/dl)	0.99	0.978–0.997	1.6
Total cholesterol (mg/dl)	0.76	0.400-0.906	3.7
Triglycerides (mg/dl)	0.82	0.544–0.929	5.8

ICC, intraclass correlation coefficient; CI, confidence interval; CV, coefficient of variation (%)

### Anthropometric characteristics

Anthropometric characteristics are displayed in Table 4. Significant main effects of time and group were observed for body mass (effect of time: *p* < 0.001, d = 1.68; effect of group: *p* = 0.002, d = 0.98), BMI (effect of time: *p* < 0.001, d = 1.70; effect of group: *p* = 0.015, d = 0.81) and body fat (effect of time: *p* < 0.001, d = 1.95; effect of group: *p* < 0.001, d = 1.70). Significant group-by- time interactions were found for body mass (*p* < 0.001; d = 1.59), BMI (*p* < 0.001; d = 1.61), and body fat (*p* < 0.001; d = 1.83). Post-hoc tests revealed a significant pre-to-post decrease for body mass and BMI in the KRTG (body mass: *p* < 0.001, d = 1.03; BMI: *p* < 0.001, d = 1.7) and BWRTG (body mass: *p* < 0.001, d = 0.44; BMI: *p* < 0.001, d = 0.56) compared to CG. Post-hoc tests also showed significant decreases in body fat variables in BWRTG (*p* < 0.001, d = 1.81) and KRTG (*p* < 0.001, d = 1.54) compared to CG. Moreover, no significant training-induced differences were observed between BWRTG and KRTG for body mass, BMI, and body fat (*p* > 0.05).

**Table 4:** Mean (± SD) values of anthropometric characteristics for the three groups

Variables	Group	Before	After	% change	*p* (Cohen’s d)		
					Main effect group	Main effect time	Interaction group x time
**Body mass (kg)**	**BWRTG**	87.25 ± 4.61	85.25 ± 4.51	-2.29	0.002 (0.98)	< 0.001 (1.68)	< 0.001 (1.59)
	**KRTG**	88.8 ± 6.60	82.7 ± 5.21	-6.87			
	**CG**	92.2 ± 6.96	91.7 ± 7.20	-0.54			
**Height (cm)**	**BWRTG**	170.75 ± 4.53	170.75 ± 4.53	0.00	---	---	---
	**KRTG**	171.95 ± 4.78	171.95 ± 4.78	0.00			
	**CG**	174.7 ± 6.45	174.7 ± 6.45	0.00			
**BMI (kg.m** ^**− 2**^ **)**	**BWRTG**	29.94 ± 1.31	29.26 ± 1.13	-2.27	0.015 (0.81)	< 0.001 (1.70)	< 0.001 (1.61)
	**KRTG**	30 ± 1.23	27.97 ± 1.16	-6.77			
	**CG**	30.18 ± 1.26	30.01 ± 1.20	-0.56			
**% Body fat (%)**	**BWRTG**	39.47 ± 0.41	36.57 ± 0.33	-7.35	< 0.001 (1.70)	< 0.001 (1.95)	< 0.001 (1.83)
	**KRTG**	39.75 ± 0.56	35.68 ± 0.91	-10.24			
	**CG**	39.71 ± 0.42	38.93 ± 0.54	-1.96			

Data are mean values (± SD), BMI: body mass index, BWRTG: body weight resistance training trained group KRTG: Kettlebell training group, CG: control group

Contrast analyses indicated that BWRTG and KRTG significantly reduced body mass (BWRTG: *p* = 0.01, d = 1.07; KRTG: *p* = 0.004, d = 1.43) and body fat (BWRTG: *p* < 0.001, d = 1.53; KRTG: *p* < 0.001 d = 1.34) compared to CG. In addition, BMI was significantly lower with KRTG (*p* = 0.004, d = 1.43) compared to CG.

### Fitness variables

Fitness variables are displayed in Table 5. Significant main effects of time were observed for VO2max (*p* < 0.001, d = 1.89), the push-up test (*p* < 0.001, d = 1.47), the sit-up test (*p* < 0.001, d = 2.00), and the sit and reach test (*p* < 0.001, d = 2.04). A significant main effect of group was observed only for VO2max (*p* < 0.001, d = 1.55). Moreover, significant group -by- time interactions were found for VO2max (*p* < 0.001, d = 1.77), the sit-up test (*p* = 0.002, d = 1), and the sit and reach test (*p* = 0.007, d = 0.87). However, there was no significant group-by-time interaction for the push-up test (*p* = 0.515, d = 0.29). For BWRTG, post-hoc tests revealed significant pre-to-post improvements for VO2max (*p* < 0.001, d = 5.47), the sit-up test (*p* < 0.001, d = 1.37), and the sit and reach test (*p* < 0.001, d = 1.66). For KRTG, significant pre-post changes were found for VO2max (*p* < 0.001, d = 1.51), the sit-up test (*p* < 0.001, d = 1.45), and the sit and reach test (*p* < 0.001, d = 1.51). Further, there were no significant training-induced differences between BWRTG and KRTG for the push-up test, the sit-up test, and the sit and reach test (*p* > 0.05).

**Table 5:** Mean (± SD) values of fitness variables for the three groups

Variables	Group	Before	After	% change	*p* (Cohen’s d)		
					Main effect group	Main effect time	Interaction group x time
**VO2max (ml/kg/min)**	**BWRTG**	28.12 ± 1.71	40.21 ± 2.62	42.99	< 0.001 (1.55)	< 0.001 (1.89)	< 0.001 (1.77)
	**KRTG**	29.07 ± 1.60	41.18 ± 2.93	41.66			
	**CG**	30.03 ± 2.22	30.83 ± 30.2	2.66			
**Push-ups (numbers)**	**BWRTG**	19.1 ± 1.37	20.45 ± 1.43	7.07	0.401 (0.35)	< 0.001 (1.47)	0.515 (0.29)
	**KRTG**	19.15 ± 2.18	20.90 ± 1.41	9.14			
	**CG**	18.90 ± 1.86	19.95 ± 1.76	5.56			
**Sit-ups (numbers)**	**BWRTG**	22.85 ± 2.30	25.4 ± 1.27	11.16	0.797 (0.20)	< 0.001 (2)	0.002 (1.0)
	**KRTG**	22.95 ± 1.82	25 ± 1.17	8.93			
	**CG**	23.55 ± 1.64	24.1 ± 1.52	2.34			
**Sit and reach (cm)**	**BWRTG**	22.85 ± 1.76	25.4 ± 1.27	11.16	0.368 (0. 35)	< 0.001 (2.04)	0.007 (0.87)
	**KRTG**	21.95 ± 2.61	25 ± 1.17	13.90			
	**CG**	23.2 ± 2.28	24.1 ± 1.52	3.88			

Data are mean values (± SDs), BWRTG: body weight resistance training trained group KRTG: Kettlebell training group, CG: control group

Contrast analyses indicated that only VO2max was significantly reduced with BWRTG (*p* < 0.001, d = 1.32) and KRTG (*p* < 0.001, d = 1.34) compared to CG.

### Physiological variables

Physiological variables are displayed in Table 6. Significant main effects of time and group were observed for vital capacity (effect of time: *p* < 0.001, d = 3.19; effect of group: *p* = 0.012, d = 0.82), resting heart rate (effect of time: *p* < 0.001, d = 2.13; effect of group: *p* = 0.013, d = 0.81), breath holding time (effect of time: *p* < 0.001, d = 3.05; effect of group: *p* < 0.001, d = 1.28), and respiratory rate (effect of time: *p* < 0.001, d = 1.71; effect of group: *p* < 0.001, d = 1.15). Significant group-by-time interactions were found for vital capacity (*p* < 0.001, d = 1.44), resting heart rate (*p* < 0.001, d = 1.29), breath holding time (*p* < 0.001, d = 1.69), and respiratory rate (*p* < 0.001, d = 1.49). However, no significant group -by-time interactions (*p* = 0.133, d = 0.54), main effects of time (*p* = 0.884, d = 0) or group (*p* = 0.736, d = 0.21) were found for mean arterial blood pressure.

**Table 6:** Mean (± SD) values of physiological variables for the three groups

Variables	Group	Before	After	% change	*p* (Cohen’s d)		
					Main effect group	Main effect time	Interaction group x time
**Vital capacity (mL)**	**BWRTG**	2919 ± 476.07	3725 ± 389.16	27.61	0.012 (0.82)	< 0.001 (3.19)	< 0.001 (1.44)
	**KRTG**	3057.5 ± 308.34	4035 ± 391.59	31.97			
	**CG**	3072.5 ± 451.16	3330 ± 478.04	8.38			
**Resting heart rate (bpm)**	**BWRTG**	75.2 ± 3.74	69.95 ± 3.82	-6.98	0.013 (0.81)	< 0.001 (2.13)	< 0.001 (1.29)
	**KRTG**	73.9 ± 3.51	70.8 ± 4.25	-4.19			
	**CG**	75.65 ± 3.01	74.9 ± 3.51	-0.99			
**Mean arterial blood pressure (mmHg)**	**BWRTG**	97.93 ± 2.61	96.76 ± 2.36	-1.19	0.736 (0.21)	0.884 (0.00)	0.133 (0.54)
	**KRTG**	97.98 ± 2.59	97.88 ± 2.44	-0.10			
	**CG**	96.96 ± 3.70	98.46 ± 4.57	1.55			
**Breath holding time (s)**	**BWRTG**	35.85 ± 3.76	54.5 ± 8.91	52.02	< 0.001 (1.28)	< 0.001 (3.05)	< 0.001 (1.69)
	**KRTG**	36.9 ± 4.38	51.35 ± 8.77	39.16			
	**CG**	36.95 ± 3.98	39.85 ± 5.45	7.85			
**Respiratory rate (numbers)**	**BWRTG**	33.3 ± 3.33	27.25 ± 3.39	-18.17	< 0.001 (1.15)	< 0.001 (1.71)	< 0.001 (1.49)
	**KRTG**	32 ± 4.23	28.8 ± 3.46	-10.00			
	**CG**	34.2 ± 4.48	34.5 ± 3.90	0.88			

Data are mean values (± SD), BWRTG: body weight resistance training trained group KRTG: Kettlebell training group, CG: control group

Post-hoc tests revealed significant pre-to-post improvements for vital capacity (*p* < 0.001; d = 1.85) and breath holding time (*p* < 0.001; d = 2.73) for BWRTG. With regards to KRTG, significant pre-post improvements were found for vital capacity (*p* < 0.001; d = 1.28) and breath holding time (*p* < 0.001; d = 2.09). In addition, post-hoc tests revealed significant pre-to-post decreases for both, BWRTG and KRTG compared to CG for the parameters resting heart rate (BWRTG: *p* < 0.001, d = 1.38; KRTG: *p* = 0.001, d = 0.8) and respiratory rate (BWRTG: *p* < 0.001, d = 1.8; KRTG: *p* < 0.001, d = 0.83). In addition, no significant training-induced differences were observed between BWRTG and KRTG for vital capacity and breath holding time, resting heart rate, and respiratory rate (*p* > 0.05).

Contrast analyses indicated that vital capacity was significantly improved with KRTG compared to CG (*p* < 0.001, d = 1.61) and BWRTG (*p* = 0.004, d = 0.79). Also, contrast analyses highlighted a significant decrease in both, KRTG and BWRTG compared to CG for resting heart rate (KRTG: *p* = 0.024, d = 1.05; BWRTG: *p* = 0.041, d = 1.35) and respiratory rate (KRTG: *p* = 0.001, d = 1.55; BWRTG: *p* = 0.001, d = 1.98). However, contrast analyses indicated that vital capacity significantly improved with KRTG compared to CG (*p* = 0.011, d = 1.61), and that breath holding time significantly improved with KRTG (*p* = 0.001, d = 1.58) and BWRTG (*p* < 0.001, d = 1.98) compared to CG.

### Biochemical variables

Biochemical variables are displayed in Table 7. Significant main time effects were observed for high-density lipoprotein (*p* < 0.001, d = 1.47), low-density lipoprotein (*p* < 0.001, d = 1.92), total cholesterol (*p* < 0.001, d = 1.98), and triglycerides (*p* = 0.009, d = 0.71). Significant group-by-time interactions were found for total cholesterol (*p* < 0.001, d = 1.59) and low density lipoprotein (*p* < 0.001, d = 1.82). Moreover, significant main effects of group were observed for total cholesterol (*p* = 0.007, d = 0.87) and low-density lipoprotein (*p* = 0.005, d = 0.90). Post-hoc tests revealed significant pre-to post decreases for total cholesterol (*p* < 0.001, d = 1.42) and low density lipoprotein (*p* < 0.001, d = 1.60) in BWRTG, and in KRTG (*p* < 0.001, d = 1.63), d = 1.85) compared to CG. Moreover, no significant training-induced differences were observed between BWRTG and KRTG for total cholesterol.

**Table 7:** Mean (± SD) values of biochemical variables for the three groups

Variables	Group	Before	After	% change	*p* (Cohen’s d)		
					Main effect group	Main effect time	Interaction group x time
**High Density Lipoprotein (HDL) (mg/dl)**	**BWRTG**	53.95 ± 3.83	55.6 ± 2.14	3.06	0.580 (0.28)	< 0.001 (1.47)	0.304 (0.41)
	**KRTG**	52.60 ± 3.07	55.80 ± 2.07	6.08			
	**CG**	53.85 ± 2.96	55.95 ± 2.09	3.90			
**Low Density Lipoprotein (LDL) (mg/dl)**	**BWRTG**	126.27 ± 8.39	112.84 ± 8.44	-10.64	0.005 (0.90)	< 0.001 (1.91)	< 0.001 (1.82)
	**KRTG**	120.46 ± 6.56	107.89 ± 7.01	-10.43			
	**CG**	123.49 ± 9.98	122.96 ± 10.38	-0.43			
**Total cholesterol (mg/dl)**	**BWRTG**	216.8 ± 7.13	204.3 ± 10.24	-5.77	0.007 (0.87)	< 0.001 (1.98)	< 0.001 (1.59)
	**KRTG**	213.07 ± 6.78	200.22 ± 8.84	-6.03			
	**CG**	214.12 ± 6.83	215.2 ± 11.57	0.50			
**Triglycerides (mg/dl)**	**BWRTG**	181.52 ± 11.31	179.3 ± 10.24	-1.22	0.793 (0.18)	0.009 (0.71)	0.417 (0.35)
	**KRTG**	183.89 ± 7.88	180.2 ± 10.10	-2.01			
	**CG**	180.35 ± 11.79	179.42 ± 13.46	-0.52			

Data are mean values (± SD), BWRTG: body weight resistance training trained group KRTG: Kettle Bell trained group, CG: control group

Contrast analyses indicated that low-density lipoprotein significantly decreased only with KRTG (*p* = 0.004, d = 1.70) compared to CG. Moreover, contrast analyses highlighted a significant decrease of total cholesterol with only KRTG (*p* = 0.005, d = 1.45) compared to CG.

## Discussion

This study compared the effects of 12 weeks of BWRTG versus KRTG and passive control on physical fitness and physiological responses including blood lipid profiles of obese young adult males. The main results of this study were that both, KRTG and BWRTG improved selected measures of physical fitness such as cardiorespiratory fitness (12-minute Cooper run test), muscular endurance (sit-up test), flexibility (sit and reach test) as well as body composition (i.e., reduction in body mass, body fat, BMI). Additionally, a significant increase in vital capacity, total apnea time, also including a significant decrease in cholesterol levels and low-density lipoprotein levels were found after 12 weeks of BWRTG and KRTG. However, no statistically significant differences in training-induced adaptations were observed between KRTG and BWRTG for all other parameters.

In terms of anthropometrics, it is evident that both BWRTG and KRTG are effective interventions to improve body mass, and body fat percentage except BMI (only with KRTG). These results align with the current body of the literature, which consistently supports the benefits of exercise on various health parameters [38]. The significant reductions in body mass and BMI for both BWRTG and KRTG can be attributed to the increased energy expenditure during exercise [39], leading to a negative energy balance and subsequent body mass loss [40]. Previous studies have also demonstrated the effectiveness of BWRTG for reducing body mass and the BMI, as it enhances cardiovascular fitness, muscular endurance, and overall physical capacity [41]. BWRTG involves performing exercises that mimic real-life movements and engage multiple muscle groups simultaneously. BWRTG has been shown to improve physical function and overall performance in various populations, including older adults with chronic conditions. Resistance training in general has been found to improve physical function and measured physical performance in older adults with coronary heart disease [42] and older individuals with Parkinson’s disease [43]. Moreover, resistance training, including BWRTG types, can enhance muscular endurance and strength development in children [41] and has been shown to be safe and effective for children, leading to improvements in muscular strength and endurance [41]. On the other hand, KRTG, a specific type of resistance training, has been growing in popularity due to its potential to improve physical fitness [44]. The observed reduction in body mass, the BMI, and body fat percentage in the KRTG supports existing evidence that highlights the benefits of resistance training for promoting body mass loss and improving body composition [45]. Interestingly, the contrast analysis revealed significant differences between the KRTG and BWRTG only for body fat percentage reduction. This suggests that while both exercise interventions led to improvements in body mass and the BMI, KRTG may be more effective in reducing body fat. This finding can be supported by the known role of resistance training in stimulating muscle hypertrophy, which in turn increases resting metabolic rate and promotes greater fat loss [46].

With respect to fitness variables, our results demonstrate significant improvements in VO2max, the sit-up test, and the sit and reach test for both intervention groups, with no significant differences between the two training groups. The improvements in VO2max observed in BWRTG and KRTG contribute to the existing literature that highlights the benefits of different exercise modalities on cardiorespiratory fitness [47, 48]. The enhancements in VO2max achieved through both BWRTG and KRTG mirror the outcomes often associated with traditional aerobic exercise programs, such as running or cycling, which are well-known for their efficacy in improving cardiorespiratory fitness. However, it is noteworthy that the significant VO2max improvements from resistance-oriented modalities like kettlebell and bodyweight training highlight their potential as viable alternatives to conventional aerobic exercises, particularly for individuals who may prefer or require variety in their fitness routines. Furthermore, it is worth mentioning that the enhanced cardiorespiratory fitness has been associated with a reduced risk of sustaining cardiovascular diseases, improved quality of life, and better physical fitness [49]. The observed improvements in sit-ups and sit and reach tests in both the BWRTG and KRTG underscore the importance of incorporating exercises that target trunk muscle strength and flexibility exercises in a well-rounded fitness program [50, 51]. Trunk muscle strength plays a critical role in maintaining proper posture and minimizing the risk of injuries, while flexibility is essential for maintaining a functional range of motion and reducing musculoskeletal discomfort [52, 53]. Although no significant group-by-time interaction was observed for the push-ups test, it remains essential to consider that various factors, such as individual differences in upper body strength and technique, could have influenced the results [54].

Regarding physiological variables, both the BWRTG and KRTG showed significant improvements in vital capacity, resting heart rate, breath holding time, and respiratory rate compared to the CG. The observed improvements in vital capacity, resting heart rate, breath holding time, and respiratory rate for both the body weight resistance training group and kettlebell training group are in line with previous research demonstrating the beneficial effects of exercise on various physiological parameters [39, 55]. These improvements can be attributed to the adaptations of the cardiovascular and respiratory systems to regular exercise, which in turn result in better overall health and physical fitness [56, 57]. An enhanced vital capacity indicates a higher efficiency of the respiratory system and has been linked to increased lung function and aerobic capacity [57]. The finding that vital capacity improved more significantly in the KRTG compared to both the CG and the BWRTG suggests that KRTG may have a more pronounced effect on lung function, possibly due to the involvement of both aerobic and anaerobic components in kettlebell exercises [58]. The observed reductions in resting heart rate and respiratory rate in both the BWRTG and KRTG are consistent with previous studies, which have reported that regular exercise can lower resting heart rate, reflecting increased cardiac efficiency, and reduce respiratory rate, indicating more efficient oxygen utilization [59, 60]. These improvements contribute to a reduced risk of cardiovascular diseases and better overall health [61]. The enhancement in breath holding time in both the BWRTG and KRTG is indicative of improved respiratory muscle strength and efficiency [62]. Indeed, increased breath holding time has been associated with better lung function, increased tolerance to high-intensity exercise, and improved performance in various sports [63].

The results regarding the biochemical variables assessed in this study showed significant improvements in high-density lipoprotein, low-density lipoprotein, and total cholesterol following both BWRTG and KRTG interventions. The observed improvements in high-density lipoprotein, low-density lipoprotein, and total cholesterol levels in the study align with previous research demonstrating the positive effects of exercise on lipid profiles [64, 65]. Regular physical activity has been associated with favourable changes in these biochemical markers, which in turn may contribute to a reduced risk of cardiovascular disease [66, 67]. The findings of this study are consistent with the well-documented benefits of BWRTG on lipid metabolism [68]. It is noteworthy that KRTG also showed significant improvements in LDL and total cholesterol levels. This supports the growing body of evidence that resistance training can provide similar benefits to BWRTG in modulating lipid profiles [69]. The lack of a significant group-by-time interaction for HDL may suggest that both training modalities have comparable effects on this parameter. However, it is important to consider that the response to exercise interventions can be influenced by factors such as baseline lipid levels, genetic predisposition, and dietary intake, which could potentially affect the observed results [70].

The present study demonstrated that both BWRTG and KRTG led to significant improvements in anthropometric, physiological, physical fitness, and biochemical variables compared to the control group. However, some differences were observed between the two training modalities, with BWRTG showing greater effects on body fat reduction and KRTG being more effective in improving vital capacity and total cholesterol levels. These findings suggest that a combination of both training modalities may be beneficial in optimizing health and fitness outcomes in young male obese individuals.

### Limitations

Considering the study’s limitations, more research is required to examine the associations between morphological parameters and physical, physiological, and biological variations, which will create the basis for future research. Indeed, while the emphasis of our study was on the impact of BWRTG or KRTG on measures of physical fitness related to health, physiological and blood lipid profiles could further and clarify our results. Similarly, future studies are needed to investigate the effects of aerobic training and kettlebell training on physical and physiological variations in obese female subjects. Additionally, future research should consider the importance of dietary control in obese individuals to optimize dietary interventions with resistance training using a multimodal approach.

### Practical applications

The practical implications of our study findings are relevant for fitness professionals and healthcare providers interested in optimizing exercise interventions for body composition improvement and overall health enhancement in obese individuals. As both BWRTG and KRTG demonstrated significant reductions in body mass, BMI, and body fat, it is evident that incorporating either exercise modality into a regular exercise routine can be effective in promoting positive changes in body composition. This insight can be valuable for fitness professionals when designing tailored exercise programs for clients with body fat reduction goals. Moreover, healthcare providers can use this information to make evidence-based recommendations for patients seeking to improve their body composition and overall health, particularly for those with obesity-related comorbidities.

## Conclusions

In conclusion, this study examined the effects of two different resistance training modalities (BWRTG, KRTG) on measures of physical fitness, body composition, as well as physiological and biochemical variables in obese adults. Both exercise modalities resulted in selected improvements in physical fitness, body composition, and certain physiological and biochemical markers. Accordingly, either resistance training type can be incorporated into a regular exercise routine.

It is essential to consider individual goals, preferences, and physical abilities when tailoring exercise interventions for optimal results. While this study highlights the effects of two resistance training modalities for body fat reduction, other factors, such as enjoyment and adherence, should also be taken into account when selecting the most appropriate exercise modality. Future research should explore the potential synergistic effects of combining aerobic and resistance training, as well as investigate optimal training frequencies, durations, and intensities for various populations and health outcomes. Ultimately, the findings of this study contribute to the evidence base supporting the role of exercise in promoting improved body composition, fitness, and overall health in obese adults.

### Electronic supplementary material

Below is the link to the electronic supplementary material.

Supplementary Material 1

## Data Availability

The datasets generated during and analyzed during the current study are not publicly available due to confidential information about the participants but are available from the corresponding author on reasonable request.
